# Impacts of multicollinearity on CAPT modalities: An heterogeneous machine learning framework for computer-assisted French phoneme pronunciation training

**DOI:** 10.1371/journal.pone.0257901

**Published:** 2021-10-18

**Authors:** Yanjing Bi, Chao Li, Yannick Benezeth, Fan Yang

**Affiliations:** 1 School of Foreign Studies, Capital University of Economics and Business, Beijing, China; 2 Institute of Acoustics, Chinese Academy of Sciences, Beijing, China; 3 University of Chinese Academy of Sciences, Beijing, China; 4 Laboratory ImViA, Université Bourgogne Franche-Comté, Dijon, Burgundy, France; Newcastle University, UNITED KINGDOM

## Abstract

Phoneme pronunciations are usually considered as basic skills for learning a foreign language. Practicing the pronunciations in a computer-assisted way is helpful in a self-directed or long-distance learning environment. Recent researches indicate that machine learning is a promising method to build high-performance computer-assisted pronunciation training modalities. Many data-driven classifying models, such as support vector machines, back-propagation networks, deep neural networks and convolutional neural networks, are increasingly widely used for it. Yet, the acoustic waveforms of phoneme are essentially modulated from the base vibrations of vocal cords, and this fact somehow makes the predictors collinear, distorting the classifying models. A commonly-used solution to address this issue is to suppressing the collinearity of predictors via partial least square regressing algorithm. It allows to obtain high-quality predictor weighting results via predictor relationship analysis. However, as a linear regressor, the classifiers of this type possess very simple topology structures, constraining the universality of the regressors. For this issue, this paper presents an heterogeneous phoneme recognition framework which can further benefit the phoneme pronunciation diagnostic tasks by combining the partial least square with support vector machines. A French phoneme data set containing 4830 samples is established for the evaluation experiments. The experiments of this paper demonstrates that the new method improves the accuracy performance of the phoneme classifiers by 0.21 − 8.47% comparing to state-of-the-arts with different data training data density.

## 1 Introduction

Within linguistic theories, phonemes play a central role as units of speech perception and access codes to lexical representations, and phoneme pronunciations are usually considered as basic skills for learning a foreign language. Computer-assisted pronunciation training (CAPT) is helpful for pronunciation practice and mispronunciation identification in a self-directed or long-distance learning environment. Conventional CAPT modalities offer verification feedbacks via automatic acoustic analysis. Because phonemes are essentially ‘segment-sized’ (have different sizes in time domain) and abstract (have different acoustic realisations), identifying the phonemes of speeches by using some physical model can hardly satisfy the requirements of today’s CAPT applications.

Recently, machine learning (ML) techniques have made great progresses, providing new opportunities to update CAPT modalities [1–8]. From the view point of computer sciences, phoneme pronunciation diagnostics are naturally target classification tasks, so we can benefit from the advances of regression analysis methods, which address the classification issues by making data-driven predictions or decisions through building a statistical model from the recorded speech data instead of analytical functions. Piotrowska et al. [1] use the parameterized audio vector as the feature vectors to improve the automatic allophone classifier, and it is reported that this method achieves an accuracy performance of 98% in the dark and clear [l] distinguishing tasks. Almajai et al. [6] develop a Deep Learning based speaker-independent speech recognizing method, which possess better accuracy performance than the conventional methods such as linear regression and maximum likelihood linear transform in the comparative evaluations. Brocki and Marasek [8] propose a DBNN-BLSTM hybrid acoustic model for large vocabulary continuous speech recognitions by combining the deep belief neural network with bidirectional long-short time memory (BLSTM) hybrid. This new method improve the recognition rate by 5% comparing to the classical BLSTM method in the low-corpus-size speech recognition tasks. Abdel-Hamid et al. [4] improve the convolutional neural network (CNN) via limited-weight-sharing scheme and use it to speech recognitions. Experiments show that it reduces the the error rate by 6–10% compares with conventional deep neural networks (DNNs) on the TIMIT phone recognition and the voice search large vocabulary speech recognition tasks. Zehra et al. [9] experimentally investigate the ensemble learning effect using a majority voting technique for cross-corpus, multi-lingual speech emotion recognition system, proving that this approach gives promising results against other state-of-the-art techniques.

However, the models of this type are sensitive to mullticollinearity of the predictors, always resulting in model distortions [10]. The multicollinearity problem means that one of the predictor variables in a classification model can be linearly predicted from the others with a substantial degree of accuracy. A set of variables is perfectly collinear if one or more exact linear relationships exists among some of the variables:

$$\begin{array}{c} x_{0}+q_{1}x_{1}+q_{2}x_{2}+\cdots +q_{i}x_{i}=0 \end{array}$$ (1)

where *q*_*i*_ is constant corresponding to the *i*-th predictor *x*_*i*_. Although it is usually difficult to figure out a precise mathematical model to explain the fundamentals in a certain pattern recognition problem, many researches indicate that suppressing the multicollinearity by using some suitable method is helpful to improve the pattern discriminability. For example, Nguyen and Rocke [11] adapt partial least squares to reduce the sample vector dimensions in the analysis procedure of human tumor sample classifications based on microarray gene expressions. Uzair et al. [12] develop a hyperspectral face recognition application in the biometric field, which effectively improve the test accuracy by modeling the relations between training and prediction matrices. Li et al. [13] incorporate multicollinearity suppressing cycle into the multi-spectrum palmprint recognition framework and achieve a very high recognition rate nearly 100%.

**Fig 1:**
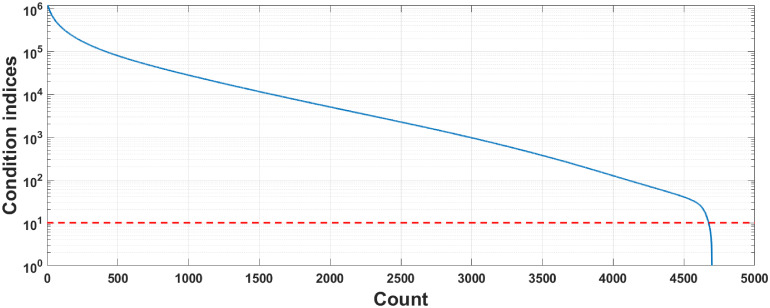
Condition indices of phoneme frequency spectral.

Similarly, phoneme utterance diagnoses may also face the issue of multicollinearity problem, which is never explored in the field of CAPT. Because the utterances are made from base vibrations of vocal cords through resonance chambers (buccal, nasal and pharyngeal cavities) [14, 15], the predictors of the phoneme feature vectors are highly probably collinear. Our quantitative diagnose results demonstrate the multicollinearity problem of utterances by using condition indices (CIs) [16]:

$$\begin{array}{c} CI_{i}=\sqrt{\frac{\lambda_{max}}{\lambda_{i}}} \end{array}$$ (2)

where λ_*max*_ is the maximum eigenvalue of the symbol vector, and λ_*i*_ is its *i*-th eigenvalue. Belsey et al. [17] suggest that predictor dependencies start to affect the regression estimates when the CI is higher than 10. Fig 1 plots the condition indices of a phoneme frequency spectrum set, in which 87.27% of the elements exceed this suggested threshold line.

The work of this paper focuses on the French CAPT. It is motivated by the fact that the existing research findings demonstrate that ML-based CPAT modalities are usually distorted by the predictor collinearity. We therefore attempt to improve their accuracy performance by mitigating this problem. To do this, with the help of 23 volunteers, a new phoneme database, namely CUEB French Phoneme Database 1.0, is first established. It contains 35 phonemes ×6 sessions ×23 persons = 4830 samples, allowing us to verify the relevant theories or hypothesis. Next, as shown in Fig 1, the multicollinearity of the French phoneme utterances is analyzed, and the results indicate that it indeed exists in the case of this paper and plays a role of negative influencing factor. Thirdly, according to a state-of-art review, the partial least square (PLS) regression algorithm is used to suppress the multicollinearity of utterance sample vectors. The evaluation results show that it is an effective solution for this issue, but it is also found that the accuracy of the PLS-only classifies are lower than the typical machine learning models, i.e. support vector machines (SVMs) and DNNs. Hence, we incorporate the improved soft-margin SVMs into the target CAPT modality in order to further enhance its feature recognition ability. Finally, an heterogeneous ML framework for French phoneme pronunciation recognition is implemented and evaluated by comparing with four state-of-the-arts: PLS-only regressors, hard-margin SVM, soft-margin SVM and DNN. The innovations of this work include:

1. The multicollinearity problem of the phoneme utterances are quantitatively analyzed and experimentally verified. Up to our knowledge, this is the first time that the phoneme utterance diagnostic problem is investigated from the view point of this theory.
2. A new heterogeneous ML framework for French phoneme pronunciation recognition is proposed. More precisely, the PLS regression algorithm is first used on the frequency spectrum of phoneme waveforms in order to suppress their multicollinearity, then the exacted features are classified via improved soft-margin SVMs. As a result, the accuracy performance of the target French CAPT modality is improved by 0.21 − 8.47% comparing to the state-of-the-arts with different data training data density.
3. A new CUEB French Phoneme Database is established. This database contains thousands of high-quality French phoneme utterance samples collected from 23 French teachers and learners, so can be used as a nice test bench in the future works.

The remainder of this paper is organized as follows: Section 2 describes the proposed CAPT framewrk; Section 3 presents the training process of the phoneme classification model; Section 4 analyzes the evaluation experiment results; finally, a conclusion is given in Section 5.

## 2 Proposed CAPT framework

**Fig 2:**
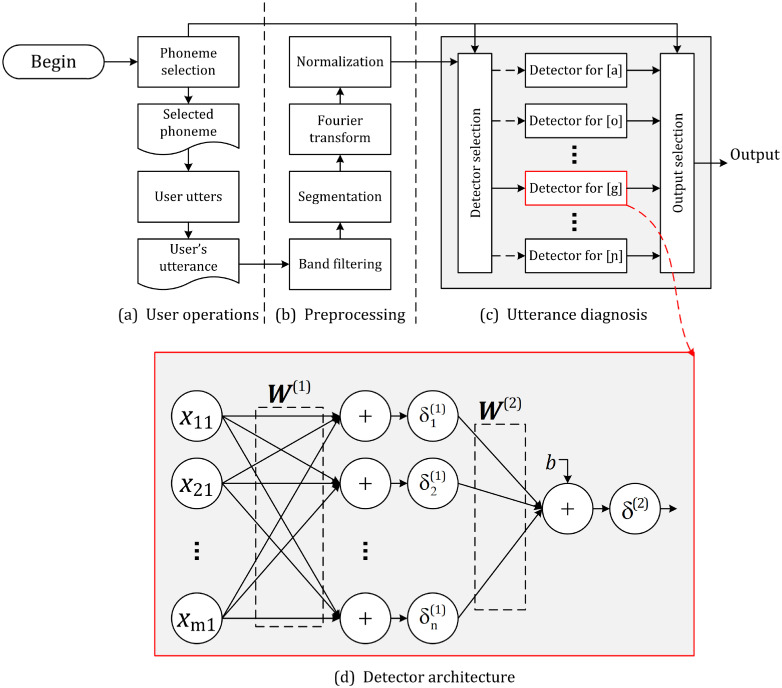
Proposed French CAPT framework.

**Fig 3:**
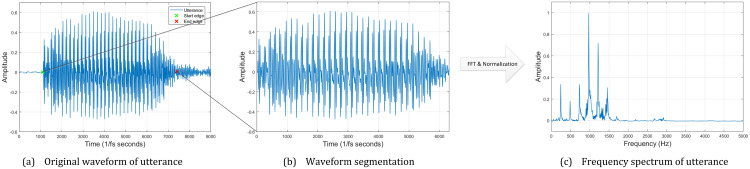
Preprocessing of an utterance example of vowel [ɑ].

Fig 2 shows the overall framework of the proposed CAPT. Users utter the phoneme to learn and record it as the input of the system. The input utterance is first filtered via a band-pass filter for denoising. Fig 3(a) plots a waveform example of vowel [ɑ]. Next, the waveform is segmented as follows:

$$tic=\{\begin{array}{ll} t & \text{if}\;P(t)>\eta_{tic}\\ nan & \text{otherwise} \end{array}$$ (3)

and

$$toc=\{\begin{array}{ll} t & \text{if}\;P(t)<\eta_{toc}\\ nan & \text{otherwise} \end{array}$$ (4)

where *tic* and *toc* are the start and end edge of the segmentation of interests, respectively. *t* is time, *P*(*t*) is instantaneous power. *η*_*tic*_ and *η*_*toc*_ are two user-defined power threshold values. Fig 3(b) zooms in the segmenting result of the given waveform. Finally, the frequency spectrum of the segmentation of interest F is computed via Fourier Transform. As shown in Fig 3(c), the normalized frequency spectrum is used as the predictor vector of detectors:

$$\begin{array}{c} x=\frac{\left|F\right|-\bar{F}}{\Delta } \end{array}$$ (5)

where $\bar{F}$ is the mean of the vector F, and Δ is the difference between its maximum and minimum values.

Finally, the predictor vector ***x*** is assigned to the corresponding detector depending on the user-selected phoneme for diagnosis. As shown in Fig 2(c), a single detector is trained specially for every phoneme. Fig 2(d) displays the architecture of the detector unit, and we can see that it is a 2-layer network architecture whose output *y* can be mathematically described as

$$\begin{array}{c} y=\delta^{\left(2\right)}\left(h^{\left(2\right)}\left(\delta^{\left(1\right)}\left(h^{\left(1\right)}\left(x^{\left(1\right)}\right)\right)\right)\right) \end{array}$$ (6)

Within Eq 6, *h*^(1)^ and *h*^(2)^ are the propagation functions of the first and second layers, whereas *δ*^(1)^ and *δ*^(2)^ are two activation function sets. More precisely, we have *h*^(1)^ and *h*^(2)^ as

$$\begin{array}{c} h^{\left(1\right)}\left(x^{\left(1\right)}\right)=x^{\left(1\right)}\times W^{\left(1\right)} \end{array}$$ (7)

and

$$\begin{array}{c} h^{\left(2\right)}\left(x^{\left(2\right)}\right)=x^{\left(2\right)}\times W^{\left(2\right)}+b \end{array}$$ (8)

***x***^(1)^ = < *x*_11_, *x*_21_, …, *x*_*m*1_ > (*m* is the vector size) is the input of the detector, so we assign the predictor vector ***x*** to it directly. ***W***^(1)^ and ***W***^(2)^ are the coefficient matrices of the first and second layers, respectively. Their sizes are *m*-by-*n* and *n*-by-1, where *n* is the class number of the regression task of the first layer. In the case of this paper, we set *n* as 35, which is the phoneme number of the French language. *b* is the bias value of the second layer. The training methods of the coefficient matrices and bias vectors are presented in the next section. ***x***^(2)^ = < *x*_12_, *x*_22_, …, *x*_*n*2_ > is the output of the first activation function set $\delta^{\left(1\right)}=<\delta_{11}^{\left(1\right)},\delta_{21}^{\left(1\right)},\ldots ,\delta_{n1}^{\left(1\right)}>$ and its function elements are rectified linear units (ReLU):

$$\delta_{1}^{\left(1\right)}(x)=\{\begin{array}{ll} x & \text{if}\;x>0\\ 0 & \text{otherwise} \end{array}$$ (9)

We apply ReLUs to the elements of *h*^(1)^’s output vector one by one. For the output of the second layer, a sigmoid function is used as its activation function in order to constrain the output of the detector into a reasonable range from 0 to 1:

$$\begin{array}{c} \delta^{\left(2\right)}\left(x\right)=\frac{1}{1+e^{-x}} \end{array}$$ (10)

The output of the detector *y* is considered as the diagnosis score, and high score values correspond to higher utterance quality. If desired, a decision can be made via a threshold *η*. The selected phoneme is correctly pronounced if *y* > *η*, otherwise not.

## 3 Training process of the utterance detectors

As shown in Fig 2(c), the utterance diagnosis is realized by using multiple independent detectors, and every detector is specified for each French phoneme. We train the detectors through an heterogeneous process combined of partial least square (PLS) regressors and soft-margin support vector machines.

### 3.1 First layer: PLS regression

PLS is a common class of methods for modeling relations between sets of observed variables by means of latent variables. Its underlying assumption is that the observed data is generated by a system or process that is driven by a small number of latent (not directly observed or measured) variables. Its goal is to maximize the covariance between the two parts of a paired data set even though those two parts are in different spaces. That implies that PLS regression can overcome the multicollinearity problem by modeling the relationships between the predictors. Consequently, the first layer of the detector is trained via PLS regression in order to suppress the multicollinearity among the predictors.

Let x_*_ be the predictor vector of a random utterance sample for training and y_*_ its response, where * = 1, 2, …, *N*. Both of x_*_ and y_*_ are zero-mean column vectors. We present two matrices ***X*** and ***Y*** whose *i*-th rows are the predictor vectors and their responses corresponding to the *i*-th sample. Their covariance matrix ***C***_*xy*_ is given as

$$\begin{array}{c} C_{xy}=\frac{1}{N}\sum_{i=1}^{N}x_{i}y_{i}^{T}=\frac{1}{N}X^{T}Y \end{array}$$ (11)

where *N* is the utterance sample number for training.

Next, we project the predictor vectors onto two separate directions specified by unit vectors ***w***_*x*_ and ***w***_*y*_ in order to obtain two random variables: $w_{x}^{T}x_{*}$ and $w_{y}^{T}y_{*}$. According to the nonlinear iterative partial least squares algorithm, PLS searches for the directions ***w***_*x*_ and ***w***_*y*_ such that [12, 18]

$$\begin{array}{c} \begin{array}{cc} \underset{w_{x},w_{y}:||w_{x}\left|\right|=\left|\right|w_{y}\left|\right|=1}{\text{max}}C\left(w_{x},w_{y}\right) & =\underset{w_{x},w_{y}:||w_{x}\left|\right|=\left|\right|w_{y}\left|\right|=1}{\text{max}}w_{x}^{T}C_{xy}w_{y}\\ & =\underset{w_{x},w_{y}:||w_{x}\left|\right|=\left|\right|w_{y}\left|\right|=1}{\text{max}}\frac{1}{m}w_{x}^{T}X^{T}Yw_{y} \end{array} \end{array}$$ (12)

The directions that solve the maximal covariance optimization are the first singular vectors ***w***_*x*_ = ***u***_*1*_ and ***w***_*y*_ = ***v***_*1*_ of the singular value decomposition of ***C***_*xy*_ = ***U***Σ***V***^*T*^, where the value of the covariance is given by the corresponding singular value *σ*_1_. In this paper we apply the same data projecting strategy through deflation in order to obtain multiple projecting direction, and the deflation of ***X*** is written as

$$\begin{array}{c} X_{j+1}=X_{j}\left(I-u_{j}p_{j}^{T}\right) \end{array}$$ (13)

with

$$\begin{array}{c} p_{j}=\frac{X_{j}^{T}X_{j}u_{j}}{X_{j}^{T}u_{j}^{T}u_{j}X_{j}} \end{array}$$ (14)

Let *φ*(x_*_) be the feature vector of some test point. By rolling the equation above with the initialization *φ*_1_(x_*_) = *φ*(x_*_), a series of feature vectors in terms of the sample x_*_ are created:

$$\begin{array}{c} \varphi_{k+1}{\left(x_{*}\right)}^{T}=\varphi {\left(x_{*}\right)}^{T}-\sum_{j=1}^{k}\varphi_{j}{\left(x_{*}\right)}^{T}u_{j}p_{j}^{T} \end{array}$$ (15)

Compute the inner products between *φ*(x_*_) and ***u***_*i*_ stored as the columns of $\tilde{U}$:

$$\begin{array}{c} \varphi_{k+1}{\left(x\right)}^{T}\tilde{U}=\varphi {\left(x\right)}^{T}\tilde{U}-\tilde{\varphi }{\left(x\right)}^{T}P^{T}\tilde{U} \end{array}$$ (16)

with

$$\begin{array}{c} \tilde{\varphi }\left(x_{*}\right)=\varphi_{j}{\left(x\right)}^{T}u_{j} \end{array}$$ (17)

where $\tilde{\varphi }\left(x_{*}\right)$ is the feature vector needed for the regression, and ***P*** is the matrix with the columns of ***p***_*j*_ (*j* = 1, 2, 3, …, *k*). For *i* > *j*, $\left(I-u_{i}p_{i}^{T}\right)u_{j}=u_{j}$. In order to compute the regression coefficient matrix ***W***^(1)^, we seek a coefficient matrix ***B*** that solves the following optimization [19–21]:

$$\begin{array}{c} \underset{B}{\text{min}}||X\tilde{U}B-Y{\left|\right|}^{2}=\underset{B}{\text{min}}\left\langle X\tilde{U}B-Y,X\tilde{U}B-Y\right\rangle \end{array}$$ (18)

The final regression coefficients contained in ***W***^(1)^ are given by $\tilde{U}B$. We solve the optimization of Eq 18 by computing its gradient with respect to ***B***:

$$\begin{array}{c} B=\frac{\sigma_{j}v_{j}^{T}}{u_{j}^{T}X_{j}^{T}X_{j}u_{j}} \end{array}$$ (19)

where ***v***_*j*_ is the complementary singular vector associated with ***u***_*j*_ so that

$$\begin{array}{c} \sigma_{j}v_{j}=Y^{T}X_{j}u_{j} \end{array}$$ (20)

It follows that the overall regression coefficients can be computed as

$$\begin{array}{c} W^{\left(1\right)}=\tilde{U}{\left(P^{T}\tilde{U}\right)}^{-1}C^{T} \end{array}$$ (21)

where ***C*** is the matrix with columns $c_{j}=\frac{Y^{T}X_{j}u_{j}}{u_{j}X_{j}^{T}X_{j}u_{j}}$.

We train the first layer of the detector by using a training set ***X*** and its responses ***Y***^*T*^. The elements of ***Y*** are 1 if the corresponding predictor vector is matched with the user-selected phoneme and 0 otherwise. By using this way, the multicollinearity of the predictors can be well mitigated and facilitate the classifying task in the next layer.

### 3.2 Layer 2: Support vector machine

The second layer of the detector is trained by using improved soft-margin SVMs. SVM is a type of binary classifier that has been widely used [9, 22–24] in speech processing, and we write the SVM model desired in this paper as Eq 8. Classical SVMs build the classifier by searching for some hyperplane (***W***^(2)^, *b*) that maximizes the margin *γ* between the two target clusters (correct pronunciations or not):

$$\begin{array}{l} \underset{W^{\left(2\right)},b}{\text{min}}\frac{1}{2}||W^{\left(2\right)}|{|}^{2}\\ \text{s}.\text{t}.\;y_{i}(x_{i}^{\left(2\right)}\times W^{\left(2\right)}+b)\geq 1,i=1,2,\ldots ,N \end{array}$$ (22)

where $x_{i}^{\left(2\right)}$ is the *i*-th predictor vector used to train the second layer, and we have

$$\begin{array}{c} x_{i}^{\left(2\right)}=\delta^{\left(1\right)}\left(h^{\left(1\right)}\left(x_{i}\right)\right)) \end{array}$$ (23)

**Fig 4:**
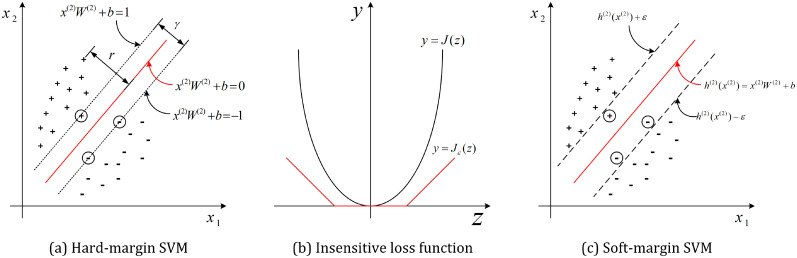
Support vector regression.

Eq 22 allows to classify the utterance samples with a “hard margin” determined by support vectors (the cycled samples in Fig 4(a)), which may result in over-fitting problem. For this issue, we regularize it to

$$\begin{array}{c} \underset{W^{\left(2\right)},b}{\text{min}}\frac{1}{2}\left|\right|W^{\left(2\right)}{\left|\right|}^{2}+C\sum_{i=1}^{N}J\left(h^{\left(2\right)}\left(x_{i}^{\left(2\right)}\right),y_{i}^{\left(2\right)}\right) \end{array}$$ (24)

where *C* is the regularization constant, and *J* is the loss function. The first term of Eq 24 $\frac{1}{2}\left|\right|W^{\left(2\right)}{\left|\right|}^{2}$ corresponds to the structural risks, whereas the second one $C\sum_{i=1}^{N}J\left(h^{\left(2\right)}\left(x_{i}^{\left(2\right)}\right),y_{i}^{\left(2\right)}\right)$ the empirical risks. This paper uses insensitive loss function *J*_*ε*_ as loss function:

$$J_{\varepsilon }(x_{i}^{\left(2\right)})=\{\begin{array}{ll} 0 & \text{if}\;|x_{\text{i}}^{\left(\text{2}\right)}|\leq \varepsilon \\ |x_{i}^{\left(2\right)}|-\varepsilon & \text{otherwise} \end{array}$$ (25)

Fig 4(b) plots the *ε*-insensitive loss function, and Eq 24 is rewritten as

$$\begin{array}{c} \underset{W^{\left(2\right)},b}{\text{min}}\frac{1}{2}\left|\right|W^{\left(2\right)}{\left|\right|}^{2}+C\sum_{i=1}^{N}J_{\varepsilon }\left(h^{\left(2\right)}\left(x_{i}^{\left(2\right)}\right)-y_{i}^{\left(2\right)}\right) \end{array}$$ (26)

where *ε* is the maximum error between the prediction results $h^{\left(2\right)}\left(x_{*}^{\left(2\right)}\right)$ and the corresponding labels $y_{*}^{\left(2\right)}$ (* = 1, 2, …, *N*). With Eq 26, the training process takes into account the loss only when the error is higher than it. That is, a 2*ε*-width margin is obtained, within which the samples (cycled in Fig 4(c)) are supposed to have been correctly classified and their losses will not be considered.

Eq 26 is solved by using the method of lagrange multiplier. To do this, two slack variables *ξ*_*i*_ and $\xi_{i}^{\prime }$ are introduced, so that

$$\begin{array}{l} \underset{W^{\left(2\right)},b,\xi_{i},\xi_{i}^{\prime }}{\text{min}}\frac{1}{2}||W^{\left(2\right)}|{|}^{2}+C\sum_{i=1}^{N}\left(\xi_{i}+{\xi^{\prime }}_{i}\right)\\ \begin{array}{ll} \text{s}.\text{t}. & h^{\left(2\right)}(x_{i}^{\left(2\right)})-y_{i}^{\left(2\right)}\leq \varepsilon +\xi_{i}\\ & y_{i}^{\left(2\right)}-h^{\left(2\right)}(x_{i}^{\left(2\right)})\leq \varepsilon +\xi_{i}^{\prime }\\ & \xi_{i}\geq 0\\ & \xi_{i}^{\prime }\geq 0 \end{array} \end{array}$$ (27)

with

$$\begin{array}{c} i=1,2,\ldots ,N \end{array}$$

The slack variables *ξ*_*i*_ and $\xi_{i}^{\prime }$ correspond to the dissatisfaction degree to the margin constraint. We write the lagrange function of Eq 27 as

$$\begin{array}{c} \begin{array}{cc} L(W^{\left(2\right)},b,\alpha ,\alpha^{\prime },\xi ,\xi^{\prime },\mu ,\mu^{\prime }) & =\frac{1}{2}\left|\right|W^{\left(2\right)}{\left|\right|}^{2}+C\sum_{i=1}^{N}\left(\xi_{i}+\xi_{i}^{\prime }\right)-\sum_{i=1}^{N}\mu_{i}\xi_{i}-\sum_{i=1}^{N}\mu_{i}^{\prime }\xi_{i}^{\prime }\\ & +\sum_{i=1}^{N}\alpha_{i}\left(h^{\left(2\right)}\left(x_{i}^{\left(2\right)}\right)-y_{i}^{\left(2\right)}-\varepsilon -\xi_{i}\right)\\ & +\sum_{i=1}^{N}\alpha_{i}^{\prime }\left(y_{i}^{\left(2\right)}-h^{\left(2\right)}\left(x_{i}^{\left(2\right)}\right)-\varepsilon -\xi_{i}^{\prime }\right) \end{array} \end{array}$$ (28)

where *μ*_*i*_ ≥ 0, $\mu_{i}^{\prime }\geq 0$, *α*_*i*_ ≥ 0 and $\alpha_{i}^{\prime }\geq 0$, which correspond to the columns of ***μ***, ***μ***′, ***α***, are the lagrange multipliers. Bring Eq 8 into 28 and compute its partial derivatives with respects to ***W***^(2)^, *b*, *ξ*_*i*_ and $\xi_{i}^{\prime }$:

$$\begin{array}{cc} & W^{\left(2\right)}=\sum_{i=1}^{N}\left(\alpha_{i}^{\prime }-\alpha \right){x_{i}^{\left(2\right)}}^{T} \end{array}$$ (29)

$$\begin{array}{cc} & \sum_{i=1}^{N}\left(\alpha_{i}-\alpha_{i}^{\prime }\right)=0 \end{array}$$ (30)

$$\begin{array}{cc} & C=\alpha_{i}+\mu_{i} \end{array}$$ (31)

$$\begin{array}{cc} & C=\alpha_{i}^{\prime }+\mu_{i}^{\prime } \end{array}$$ (32)

According to Eqs 28–32, the dual problem of Eq 27 is obtained:

$$\begin{array}{l} \underset{\alpha ,\alpha^{\prime }}{\text{max}}D(\alpha ,\alpha^{\prime })=\underset{\alpha ,\alpha^{\prime }}{\text{max}}\sum_{i=1}^{N}\left[y_{i}^{\left(2\right)}(\alpha_{i}^{\prime }-\alpha_{i})-\varepsilon (\alpha_{i}^{\prime }+\alpha_{i})\right]\\ \;-\frac{1}{2}\sum_{i=1}^{N}\sum_{j=1}^{N}\left(\alpha_{i}^{\prime }-\alpha_{i})(\alpha_{j}^{\prime }-\alpha_{j}\right)x_{i}^{\left(2\right)}{x_{j}^{\left(2\right)}}^{T}\\ \text{s}.\text{t}.\;\sum_{i=1}^{N}\left(\alpha_{i}-\alpha_{i}^{\prime }\right)=0\\ \;0\leq \alpha_{i},\alpha_{i}^{\prime }\leq C \end{array}$$ (33)

Within Eq 33, the lagrange multipliers *α*_*i*_ and $\alpha_{i}^{\prime }$ correspond to the training sample $\left(x_{i}^{\left(2\right)},y_{i}^{\left(2\right)}\right)$. For the purpose of global optima, the Karush-Kuhn-Tucker constraints must be satisfied:

$$\begin{array}{c} \left\{ \begin{array}{c} \alpha_{i}\left(h^{\left(2\right)}\left(x_{i}^{\left(2\right)}\right)-y_{i}^{\left(2\right)}-\varepsilon -\xi_{i}\right)=0\\ \alpha_{i}^{\prime }\left(y_{i}^{\left(2\right)}-h^{\left(2\right)}\left(x_{i}^{\left(2\right)}\right)-\varepsilon -\xi_{i}^{\prime }\right)=0\\ \alpha_{i}\alpha_{i}^{\prime }=0\\ \xi_{i}\xi_{i}^{\prime }=0\\ \left(C-\alpha_{i}\right)\xi_{i}=0\\ \left(C-\alpha_{i}^{\prime }\right)\xi_{i}^{\prime }=0 \end{array}\right. \end{array}$$ (34)

The constraints of Eq 34 implies that when and only when $h^{\left(2\right)}\left(x_{i}^{\left(2\right)}\right)-y_{i}^{\left(2\right)}-\varepsilon -\xi_{i}=0$ the value of *α*_*i*_ is not zero, whereas $y_{i}^{\left(2\right)}-h^{\left(2\right)}\left(x_{i}^{\left(2\right)}\right)-\varepsilon -\xi_{i}^{\prime }=0$ for that of $\alpha_{i}^{\prime }$. Additionally, it is impossible that the constraints $h^{\left(2\right)}\left(x_{i}^{\left(2\right)}\right)-y_{i}^{\left(2\right)}-\varepsilon -\xi_{i}=0$ and $y_{i}^{\left(2\right)}-h^{\left(2\right)}\left(x_{i}^{\left(2\right)}\right)-\varepsilon -\xi_{i}^{\prime }=0$ are both valid, therefore at least one of the two multipliers *α*_*i*_ and $\alpha_{i}^{\prime }$ have to be zero. Bringing Eq 29 into 8, we rewrite the propagation function of the second layer as

$$\begin{array}{c} h^{\left(2\right)}\left(x^{\left(2\right)}\right)=\sum_{i=1}^{N}\left(\alpha_{i}^{\prime }-\alpha_{i}\right)x^{\left(2\right)}{x_{i}^{\left(2\right)}}^{T}+b \end{array}$$ (35)

According to Eq 35 it can be seen that the predictor vectors making $\left(\alpha_{i}^{\prime }-\alpha_{i}\right)$ not to be zero are the support vectors, and they must be out of the *ε*-margin. Those support vectors are only parts of the training samples, so the optima of the desired SVM model is still spare.

Now we can start to compute the coefficient matrix ***W***^(2)^ with Eqs 28–33, which is actually a quadratic programming problem and can be solved by using sequential minimal optimization (SMO) method [25]. More precisely, SMO selects one or several of them for optimizing and fixes the others so that all the variables can be solved one by one. Let $\alpha_{i_{o}}$, $\alpha_{i_{o}}^{\prime }$, $\alpha_{j_{o}}$ and $a_{j_{o}}^{\prime }$ (*i*_*o*_ ≠ *j*_*o*_) be the variables to be optimized for some iteration. According to the KKT constraints of Eq 34, at least one of the two multipliers *α*_*i*_ and $\alpha_{i}^{\prime }$ have to be zero, allowing to define two of the four variables directly as zero to facilitate the optimizations. Taking $\alpha_{i_{o}}^{\prime }=0$ and $\alpha_{j_{o}}^{\prime }=0$ as an example, Eq 33 is rewritten to

$$\begin{array}{l} \underset{\alpha_{i_{o}},\alpha_{j_{o}}}{\text{max}}D(\alpha_{i_{o}},\alpha_{j_{o}},0,0)=\underset{\alpha_{i_{o}},\alpha_{j_{o}}}{\text{max}}\sum_{i\neq i_{o}}\left[y_{i}^{\left(2\right)}(\alpha_{i}^{\prime }-\alpha_{i})-\varepsilon (\alpha_{i}^{\prime }+\alpha_{i})\right]\\ \;-\frac{1}{2}\sum_{i\neq i_{o}}\sum_{j\neq j_{o}}\left(\alpha_{i}^{\prime }-\alpha_{i})(\alpha_{j}^{\prime }-\alpha_{j}\right)x_{i}^{\left(2\right)}{x_{j}^{\left(2\right)}}^{T}\\ \;+\frac{1}{2}\alpha_{i_{o}}[\sum_{j\neq j_{o}}\left(\alpha_{j}^{\prime }-\alpha_{j}\right)x_{i_{o}}^{\left(2\right)}{x_{j}^{\left(2\right)}}^{T}-2y_{i_{o}}^{\left(2\right)}-2\varepsilon ]\\ \;-\frac{1}{2}\alpha_{i_{o}}\alpha_{j_{o}}x_{i_{o}}^{\left(2\right)}{x_{j_{o}}^{\left(2\right)}}^{T}\\ \text{s}.\text{t}.\;\alpha_{i_{o}}-\alpha_{j_{o}}=c\;\text{with}\;c=-\sum_{i\neq i_{o},j_{o}}\left(\alpha_{i}-\alpha_{i}^{\prime }\right)\\ \;0\leq \alpha_{i_{o}},\alpha_{j_{o}}\leq C \end{array}$$ (36)

where the third and fourth input arguments of D correspond to $\alpha_{i_{o}}^{\prime }$ and $\alpha_{j_{o}}^{\prime }$, respectively. *c* is a constant having $\sum_{i=1}^{N}\left(\alpha_{i}-\alpha_{i}^{\prime }\right)=0$ satisfied. Solve $\alpha_{i_{o}}-\alpha_{j_{o}}=c$ for $a_{j_{o}}$ and substitute it in Eq 36:

$$\begin{array}{l} \underset{\alpha_{i_{o}}}{\text{max}}\;D(\alpha_{i_{o}},\alpha_{i_{o}}-c,0,0)=\underset{\alpha_{i_{o}}}{\text{max}}\sum_{i\neq i_{o}}\left[y_{i}^{\left(2\right)}(\alpha_{i}^{\prime }-\alpha_{i})-\varepsilon (\alpha_{i}^{\prime }+\alpha_{i})\right]\\ \;-\frac{1}{2}\sum_{i\neq i_{o}}\sum_{j\neq j_{o}}\left(\alpha_{i}^{\prime }-\alpha_{i})(\alpha_{j}^{\prime }-\alpha_{j}\right)x_{i}^{\left(2\right)}{x_{j}^{\left(2\right)}}^{T}\\ \;+\frac{1}{2}\alpha_{i_{o}}[\sum_{j\neq j_{o}}\left(\alpha_{j}^{\prime }-\alpha_{j}\right)x_{i_{o}}^{\left(2\right)}{x_{j}^{\left(2\right)}}^{T}-2y_{i_{o}}^{\left(2\right)}-2\varepsilon +cx_{i_{o}}^{\left(2\right)}{x_{j_{o}}^{\left(2\right)}}^{T}]\\ \;-\frac{1}{2}\alpha_{i_{o}}^{2}x_{i_{o}}^{\left(2\right)}{x_{j_{o}}^{\left(2\right)}}^{T}\\ \text{s}.\text{t}.\;\text{max}\{0,c\}\leq \alpha_{i_{o}}\leq \text{min}\{C,C+c\}\;\text{with}\;c=-\sum_{i\neq i_{o},j_{o}}\left(\alpha_{i}-\alpha_{i}^{\prime }\right) \end{array}$$ (37)

$D(\alpha_{i_{o}})$ is a quadratic polynomial in standard form, allowing to compute $\alpha_{i_{o}}$ by optimizing it within the domain [max{0, *c*}, min{*C*, *C*+ *c*}]. Similarly, the four multipliers $\alpha_{i_{o}}$, $\alpha_{i_{o}}^{\prime }$, $\alpha_{j_{o}}$ and $\alpha_{j_{o}}^{\prime }$ can be computed with the other hypothesises satisfying $\alpha_{i}\alpha_{i}^{\prime }=0$, including $\left\{\alpha_{i_{o}}=0,\alpha_{j_{o}}^{\prime }=0\right\}$, $\left\{\alpha_{i_{o}}^{\prime }=0,\alpha_{j_{o}}=0\right\}$ and $\left\{\alpha_{i_{o}}=0,\alpha_{j_{o}}=0\right\}$. Finally, the optimizing results with the hypothesis maximizing $D(\alpha_{i_{o}},\alpha_{j_{o}},\alpha_{i_{o}}^{\prime },\alpha_{j_{o}}^{\prime })$ are assigned to Eq 29 to compute the coefficient vector ***W***^(2)^.

According to the KKT constraints of Eq 34, for every training sample $\left(x_{i}^{\left(2\right)},y_{i}^{\left(2\right)}\right)$ it exists (*C* − *α*_*i*_)*ξ*_*i*_ = 0 and $\alpha_{i}\left(h^{\left(2\right)}\left(x_{i}^{\left(2\right)}\right)-y_{i}^{\left(2\right)}-\varepsilon -\xi_{i}\right)=0$. Therefore, if the final value of $\alpha_{i_{o}}$ is neither zero nor *C*, $\xi_{i_{o}}$ must be zero, yielding:

$$\begin{array}{c} b_{i_{o}}=y_{i_{o}}^{\left(2\right)}+\varepsilon -\sum_{j}^{N}\left(\alpha_{j}^{\prime }-\alpha_{j}\right)x_{i_{o}}^{\left(2\right)}{x_{j}^{\left(2\right)}}^{T} \end{array}$$ (38)

where $b_{i_{o}}$ is the bias value corresponding to $\left(x_{i_{o}}^{\left(2\right)},y_{i_{o}}^{\left(2\right)}\right)$. Theoretically, all the training samples satisfying 0 < *α*_*i*_ < *C* should have led to the same bias, but errors may still exist due to the data distortions. For the purpose of high robustness, the values of *b*_*i*_ are averaged so that the final bias *b* is

$$\begin{array}{c} b=\frac{1}{N}\sum_{i=1}^{N}b_{i} \end{array}$$ (39)

with

$$b_{i}=\{\begin{array}{ll} y_{i}^{\left(2\right)}+\varepsilon -\sum_{j}^{N}\left(\alpha_{j}^{\prime }-\alpha_{j}\right)x_{i}^{\left(2\right)}{x_{j}^{\left(2\right)}}^{T} & \text{if}\;0<\alpha_{i}<C\\ 0 & \text{otherwise} \end{array}$$ (40)

## 4 Experiments

This section evaluates the proposed CAPT framework. The experiments are conducted by using the CUEB French Phoneme Database 1.0. First of all, the PLS regressor is tested to see whether it can mitigate the multi-collinearity of utterance waveforms. Next, the proposed method is compared with reference pronunciation diagnostic models in order to analyze its properties. All experiments have been achieved in the environment of MATLAB.

### 4.1 Database description

**Fig 5:**
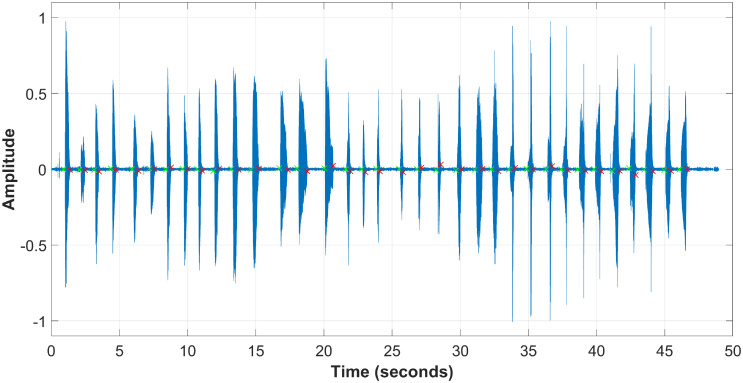
A data example of CUEB French Phoneme Database 1.0 (developed from S1 Audio).

**Fig 6:**
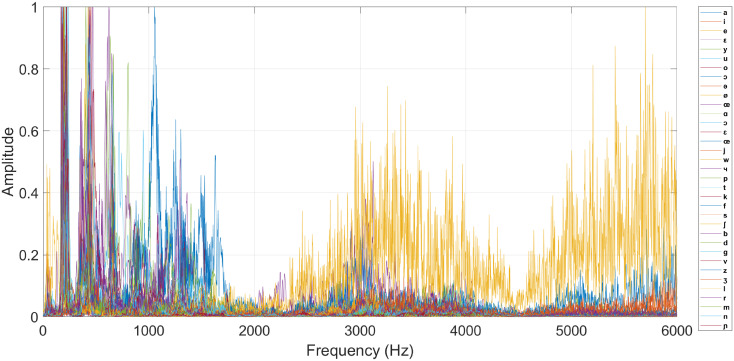
Examples of predictor vectors (developed from S1 Audio).

The CUEB French Phoneme Database 1.0 is established by the Capital University of Economics and Business and the Institute of Acoustics CAS. Within the Version 1.0, there are 23 participants, including 4 Chinese female teachers, 2 female French-native speakers, 3 Chinese male learners and 14 Chinese female learners. Every participants is asked to read the French phonemes shown in Table 1 six times to perform six different data sessions. The utterances are recorded at 44.1 kHz by using the private cell phones of the participants in a daily-life environment, further challenging the CAPT framework of this paper. Fig 5 plots an example of the recorded utterances. As presented in Fig 3, the sound waveform is segmented depending on the signal-to-noise ratio, and the threshold values *η*_*tic*_ and *η*_*toc*_ are $0.2\times \bar{P}$, where $\bar{P}$ is the mean power of the signal. The segmentation results are marked by using green and red crosses on the waveform plot, which correspond to the start and end edges, respectively. The size of the data set of this paper is therefore 35 phonemes ×6 sessions ×23 participants = 4830 samples. Fig 6 shows the examples of the predictor vectors corresponding to the 35 French phonemes, which actually are the frequency spectrum of the utterance waveforms.

**Table 1:** French phoneme table.

**15 vowels**	
Vowel:	[a], [i], [e], [ε], [y], [u], [o], [ɔ], [ə], [ø], [œ]
Nasal vowel:	[ã], [$\tilde{ɔ}$], [$\tilde{\varepsilon }$], [$\tilde{œ}$]
**3 semi vowels**	[j], [w], [ɥ]
**17 consonants**	
Deaf consonants:	[p], [t], [k], [f], [s], [ʃ]
Sound consonants:	[b], [d], [g], [v], [z], [ȝ]
Lateral consonants:	[l], [r]
Nasal consonants:	[m], [n], [ŋ]

### 4.2 Evaluation of PLS algorithm

The subject of this experiment is to evaluate the PLS algorithm of this paper. The frequency band of the signals is from 0 to 5000 Hz, and the maximum projection direction number *k* of PLS regressor is 100. The dimension of the input predictor vectors is reduced down to 500 via linear interpolation. CI values are used as the criterion to quantify the multicollinearity of the processed predictor vectors (see Eq 2). All the samples of the database are used for the measurements.

**Fig 7:**
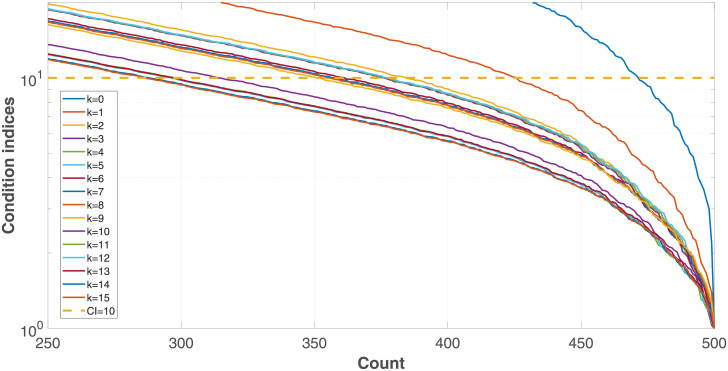
CI measurement results with different projection direction numbers.

Fig 7 plots the CI measurement results of the first 15 iterations, corresponding to the first 15 projection directions. It demonstrates that the CI values are reduced with the increase of the projection direction number *k*, indicating that the multicollinearity of the predictor vectors are mitigated step by step.

**Fig 8:**
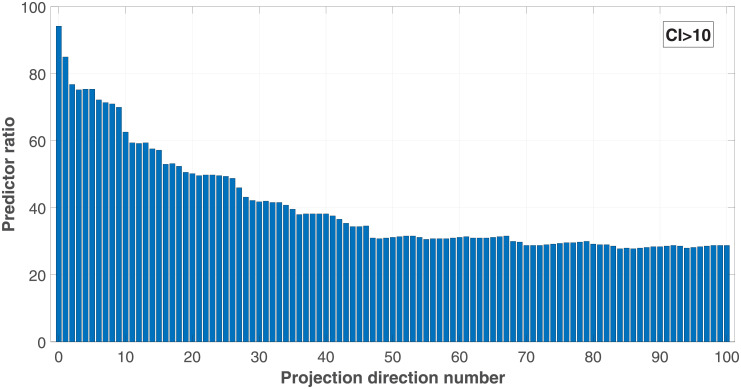
Predictor ratios at *CI* > 10 over the projection direction numbers.

The ratios of the predictors whose CI exceed 10, which is a threshold value proposed by Belsey et al. [17] for multicollinearity estimations, are computed with different projection direction numbers. The results shown in Fig 8 demonstrate also that the multicollinearity problems are mitigated, and the high-CI predictor ratio is reduced by around 64%. Meanwhile, the proposed method loss effects when *k* > 50, implying that it possess boundary effects.

### 4.3 Accuracy performance

The experiments of this subsection evaluate the accuracy performance of the proposed framework as well as its sensitivity to the PLS projection direction number *k*. 4 of the 6 sessions of the database are used to train the framework of Fig 2 whereas the rest two for testing. The curves of receiver operating characteristics (ROC) for different user-selected phonemes are measured to estimate the minimum diagnostic error rate.

**Fig 9:**
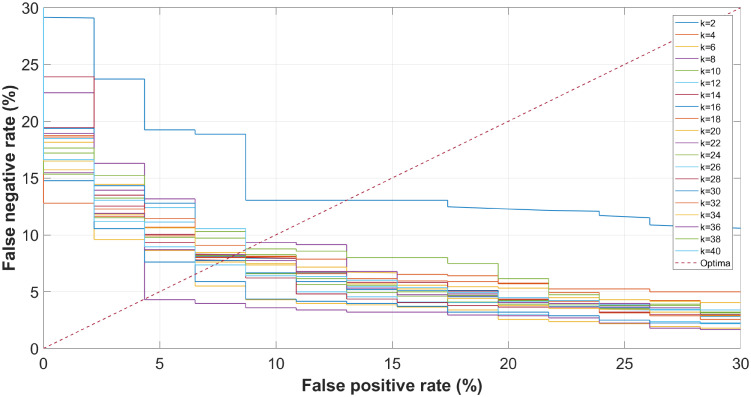
Receiver operating characteristics of the proposed CAPT framework with [ɑ].

Let us take the phoneme [ɑ] for example. When this phoneme is selected, it is actually the [ɑ]-detector of Fig 2(c) who works. Fig 9 plots its ROC curves from *k* = 2 up to *k* = 40 with a step of 2, in which x and y-axis correspond to the false positive rate (FPR) and false negative rate (FNR), respectively. The dotted line *FNR* = *FPR* is the constraint to balance the FPRs and FNRs, meaning that the minimum diagnostic error rates are obtained when the false and leak detection rates are equal. It can be seen that with the increases of projection direction, the accuracy performance of the [ɑ]-detector is improved by around 10%, implying that PLS benefits the diagnostic tasks.

**Fig 10:**
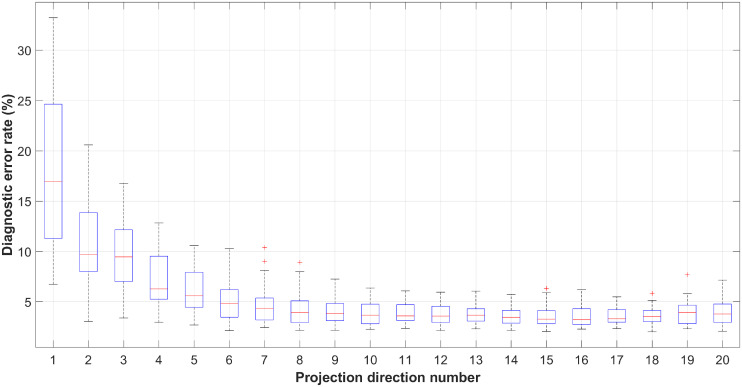
Diagnostic error rates with different PLS projection direction numbers.

**Fig 11:**
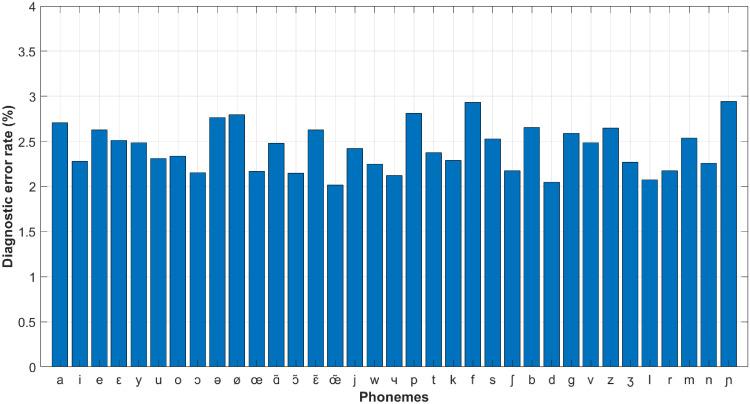
Minimum diagnostic error rates of all the phonemes: *k* ∈ [1, 100].

Applying the ROC measuring with the other phonemes we can get similar results. The minimum diagnostic error rates of all the phonemes over the PLS projection direction numbers are plotted in Fig 10, in which a single box corresponds to the diagnostic error rates of the 35 detectors measured with different projection direction numbers. On each box, the central mark indicates the median, and the bottom and top edges of the box indicate the 25th and 75th percentiles, respectively. We can see that the medians of the diagnostic error rates converge with the increase of projection direction number, demonstrating that the PLS algorithm helps to facilitate the classification tasks. Meanwhile, comparing to Fig 8, all the diagnostic error rate curves convergence after 8 iterations rather than 50, implying that the SVM classifier specified in this paper somehow has possessed the multicollinearity mitigating ability but cannot eliminate it completely. Fig 11 shows the optimal diagnostic error rates of all the 35 phonemes with *k* ∈ [1, 100], and the overall accuracy performance of the proposed CAPT framework approximates 2.43% (average minimum diagnostic error rate).

### 4.4 Comparing experiments

In order to highlight the properties of the proposed method, we compare it with the state-of-the-arts. The framework shown in Fig 2 is used as the evaluation platform. Its detectors are implemented by using the classifiers to be evaluated, including PLS regressor (PLS), hard-margin SVM (HMSVM), soft-margin (SMSVM), deep neural network (DNN) and the proposed as well.

The PLS implementation for reference is realized by using a PLS regressor which comprises of regression and classification tasks. Its final decisions are performed directly on the PLS regression without a second network layer. Its size is *m*-inputs, *m*-nodes and 1-output, where *m* is the size of the predictor vector. The HMSVM and SMSVM implementations are two classical SMV classifiers implemented with hard and soft margins, respectively (see Fig 4). Their sizes are *m*-inputs, *m*-nodes and 1-output, their maximum training iterations are 10^6^ and the minimum errors are 10^−6^. The DNN implementation is a three-hidden-layer feedforward neural network [6]. Its size is *m*-inputs, (*m* + 1)-nodes per hidden layer and 1-output, the maximum training iterations is 10^6^ and the minimum error is 10^−6^.

**Fig 12:**
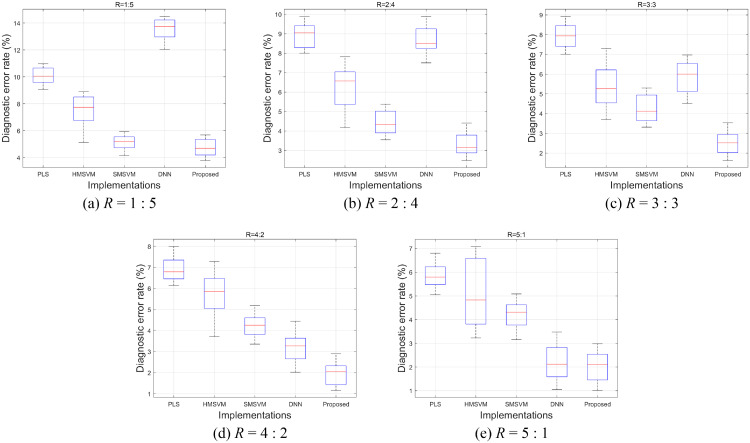
Diagnostic error rates of different implementations.

The CAPT French Phoneme database acquires 6 sessions of data from every participant, and the experiment of this subsection divide them into two groups for training and testing randomly depending on the different ratios *R* = 1: 5, 2: 4, 3: 3, 4: 2 or 5: 1. For the purpose of unbiased conclusions, the average diagnostic error rate of three measurements is used as the final evaluation result. The statistical results of the evaluation are shown in Fig 12 via box-plot. A single box corresponds to the diagnostic error rates of the 35 detectors measured with different implementations and sample ratios *R*.

The experiment results of Fig 12 demonstrate that the diagnostic error rates of all the implementations raise with the increases of the training database size. This is because providing enough training data is a well-known solution to improve the machine learning classifiers by overcoming their over-fitting problems. The median diagnostic error rates of the five implementations reduce by 4.26%, 2.91%, 0.88%, 11.62% and 2.58%, which indicate that training data sensitivity of the proposed method is lower than PLS and DNN implementations, whereas higher than the two SVM implementations. The proposed method combines the PLS and SVM methods into a single framework, so it needs a certain number of training data to find the correct data projection directions, which raise its sensitivity to the size of the training database related to SVMs. On the other hand, the SVM layers enforce the pattern classifying ability of the overall CAPT framework, allowing for lower data intensity than PLS-only or DNN implementations.

**Fig 13:**
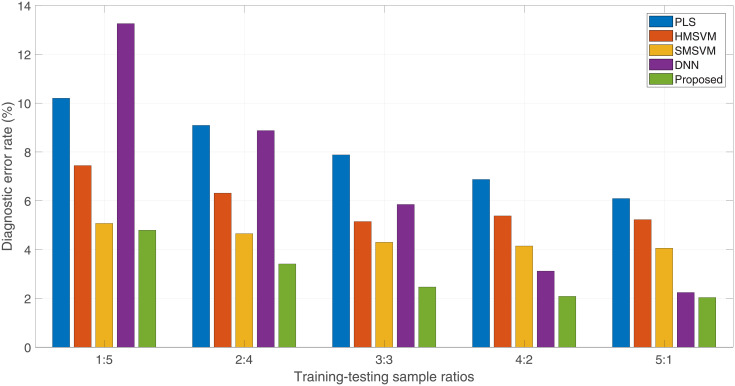
Average diagnostic error rates.

Within the experiments of this paper, the proposed heterogeneous CAPT framework achieves the best performance comparing to the reference implementations. The diagnostic error rates of the implementations are further compared in Fig 13, in which each bar indicates the average diagnostic error rate of the 35 French phoneme detectors over different training-testing sample ratios. We can see that among the reference implementations, the SMSVMs achieve the best accuracy performance at *R* = 1: 5, 2: 4 and 3: 3, whereas the DNNs at *R* = 4: 2 and 5: 1. Comparing to them, the method of this paper improves it by 0.28%, 1.24%, 1.84%, 1.03% and 0.21%. For the proposed method itself, the accuracy achievement due to the raising of sample intensity is 2.76% (*R* = 1: 5 v.s. *R* = 5: 1).

## 5 Discussions and conclusions

This paper explores the possibility to improve the ML-based French CAPT modalities via multicollinearity suppressing. Its main contributions include:

1. The assumption that the phoneme utterance recognition models of ML families are impacted by the multicollinearity problem is experimentally verified, and a PLS based solution is proposed to address it.
2. An heterogeneous machine learning framework is developed for French CAPT modalities. It combines the PLS algorithm and improved soft-margin SVM, allowing to enhancing the classifying ability of the model by mitigating the multicollinearity problem. Evaluation results show that it achieves better accuracy performance than the reference phoneme classifying models, such as PLS-only regressor, SVMs or DNNs.
3. A French phoneme database is established in order to evaluate the achievements of this work. This database contains thousands of French phoneme utterance samples collected from 23 French teachers and learners, providing a nice test bench for future works.

Despite of achievements regarding to accuracy performance within the experiments of this paper, there exists still some issues. First of all, the proposed CAPT framework is more sensitive to the data density than SVM, but less than PLS regressor and DNN. That is, it requires more data to model the relationships between the predictors for collinearity analysis than some conventional machine learning models with low-complex topology structures. Secondly, the experiment results of Fig 13 show that the performance gap between DNN and the proposed implementations gradually shrinks with the increases of data density, implying that the multi-layer networks perhaps can handle the collinearity problem under the supports of big data as well. With the constrains of data base size, we provisionally cannot make a conclusion that DNNs will lead to better performance if enough training data are provided. Conservatively speaking, the advantage of the proposed method is to allow faster convergence with sparse training data set comparing to deep learning. Therefore, the method of this paper may be more appropriate for the scenarios of data scarcity. Finally, training a classifier of this paper is time- and resource-costly, because the PLS regressing procedure is programmatically a dependent loop with low parallelism. At present, it seems hardly to embeddly realize such a model in a on-line way.

In the future work, we will attempt to further explore the collinearity-sensitivity characteristics of other ML classifiers, especially the methods of the deep learning families. PLS actually can be considered as a potential sparse-learning solution to address the data-hungry problem, which may better benefit the CAPT applications from deep learning methods.

## Supporting information

S1 Audio Phoneme pronunciation data example. An sample example of CUEB French Phoneme Database 1.0 conducting the experiments of this paper.
(M4A)

S1 Appendix (PDF)
