# Correction: Impacts of multicollinearity on CAPT modalities: An heterogeneous machine learning framework for computer-assisted French phoneme pronunciation training

**DOI:** 10.1371/journal.pone.0292513

**Published:** 2023-10-05

**Authors:** Yanjing Bi, Chao Li, Yannick Benezeth, Fan Yang

After publication of this article [1], concerns were raised that information regarding the ethical approval and participant consent information for the construction of the phoneme dataset was omitted from the article. The correct information is: The construction of the phoneme dataset was approved by the School of Foreign Studies at the Capital University of Economics and Business (Beijing, China). Participants provided written informed consent for their involvement.

The authors apologize for the error in the published article.
