# Supplementary material for: Impacts of multicollinearity on CAPT modalities: An heterogeneous machine learning framework for computer-assisted French phoneme pronunciation training
Source: PLoS One. 2021 Oct 18;16(10):e0257901. doi: 10.1371/journal.pone.0257901 (PMC8523060; doi:10.1371/journal.pone.0257901)
Supplement: S1 Appendix — (PDF) [file pone.0257901.s002.pdf]

S1 Appendix

Table 2. Notations and symbols used in this paper.

Symbols	Descriptions
CI_i	Condition index of the i-th predictor.
$\lambda_{max/i}$	The maximum/i-th eigenvalue of the symbol vector.
tic/toc	The start/end edge of the segmented utterance symbol of interests.
t	Time.
$P(t)$	Instantaneous power of the signals at t.
$\eta_{tic/toc}$	User-defined threshold value for start/end signal edge detections.
$\mathcal{F}$	Frequency spectrum vector.
$\overline{\mathcal{F}}$	Mean of frequency spectrum vectors.
Δ	The difference between maximum and minimum elements of a frequency spectrum vector.
$\boldsymbol{x}$	A predictor vector.
y	Output of the proposed CAPT network.
$h^{(1)/(2)}$	The propagation function of the first/second network layer.
$\boldsymbol{\delta}^{(1)/(2)}$	Activation function set of the first/second network layer.
$\boldsymbol{x}^{(1)/(2)}$	Input vector of the first/second network layer.
$\boldsymbol{W}^{(1)/(2)}$	the coefficient matrices of the first/second layers.
k	Projecting number of PLS regression.
b	Bias of the support vector machine model.
$\boldsymbol{X}$	Sample matrix whose rows correspond to the utterance sample vectors.
$\boldsymbol{Y}$	Response vector whose elements correspond to the observed responses.
N	Training sample number.
$\boldsymbol{C}_{xy}$	Covariance matrix of the training matrix and response vector.
FPR	False positive rate.
FNR	False negative rate.
